# Post-Vaccination Anaphylaxis in Adults: A Systematic Review and Meta-Analysis

**DOI:** 10.3390/vaccines13010037

**Published:** 2025-01-04

**Authors:** Flavia Pennisi, Anna Carole D’Amelio, Rita Cuciniello, Stefania Borlini, Luigi Mirzaian, Giovanni Emanuele Ricciardi, Massimo Minerva, Vincenza Gianfredi, Carlo Signorelli

**Affiliations:** 1 PhD National Programme in One Health Approaches to Infectious Diseases and Life Science Research, Department of Public Health, Experimental and Forensic Medicine, University of Pavia, 27100 Pavia, Italy; pennisi.flavia@hsr.it (F.P.); ricciardi.giovanni@hsr.it (G.E.R.); 2 School of Medicine, Università Vita-Salute San Raffaele, 20132 Milan, Italy; a.damelio@studenti.unisr.it (A.C.D.); borlini.stefania@hsr.it (S.B.); mirzaian.luigi@hsr.it (L.M.); minerva.massimo@hsr.it (M.M.); signorelli.carlo@hsr.it (C.S.); 3 Department of Biomedical Sciences for Health, University of Milan, Via Pascal, 36, 20133 Milan, Italy

**Keywords:** anaphylaxis, vaccines, public health, immunization, health policy

## Abstract

Background/Objectives: Vaccines have been recognized as one of the most effective public health interventions. However, vaccine-associated anaphylaxis, although rare, is a serious adverse reaction. The incidence of anaphylaxis related to non-COVID-19 vaccines in adults remains underreported. This systematic review and meta-analysis aim to estimate the incidence of post-vaccination anaphylaxis across various vaccines in adults. Methods: A comprehensive literature search of PubMed, Embase, Scopus, and Web of Science identified studies on anaphylaxis following vaccination in adults (≥18 years), excluding COVID-19 vaccines. PRISMA 2020 guidelines were followed. The protocol was registered in PROSPERO in advance (ID CRD42024566928). Random-effects and fixed-effects models were used to pool data and estimate the logit proportion, with the logit-transformed proportion serving as the effect size, thereby allowing for the calculation of event rates. Results: A total of 37 studies were included in the systematic review, with 22 studies contributing to the meta-analysis, representing a combined population of 206,855,261 participants. Most studies focused on influenza vaccines (*n* = 15). Across all studies, 262 anaphylactic cases were reported, with 153 cases related to influenza vaccines, followed by herpes zoster virus vaccines (38 cases) and yellow fever vaccines (29 cases). Td/Tdap vaccine had the lowest rate (0.0001 per 100,000 participants). The overall random-effects model yielded a logit proportion of −10.45 (95% CI: −12.09 to −8.82, *p* < 0.001), corresponding to an event rate of 2.91 events per 100,000 subjects (95% CI: 0.56 to 14.73). Sensitivity analysis showed a higher incidence for influenza, hepatitis vaccines, and in vulnerable populations. Conclusions: Anaphylaxis following vaccination in adults is rare but varies by vaccine type. Strengthened monitoring and preparedness are essential, especially in non-medical settings, to ensure a rapid response to anaphylaxis and maintain public confidence in vaccination programs.

## 1. Introduction

Anaphylaxis, as defined by the International Statistical Classification of Diseases and Related Health Problems (ICD-11) [1], is a severe, life-threatening systemic hypersensitivity reaction characterized by its rapid onset and the potential to cause fatal airway, respiratory, or circulatory complications. Although vaccines are essential in preventing infectious diseases and are generally considered safe, they can occasionally cause anaphylactic reactions [2]. The incidence of vaccine-related anaphylaxis, according to some partial estimates, ranges from 50 to 20,000 cases per 100,000 person-years [3]. Fatal outcomes are exceedingly rare, with an estimated occurrence of approximately 0.05 cases per 100,000 people annually [4].

The timely recognition and management of anaphylaxis are essential for patient safety and maintaining public trust in vaccination programs [5]. Moreover, because IgE-mediated reactions are reproducible upon re-exposure, a severe allergic reaction to a vaccine is considered a contraindication to future doses of the same vaccine [6].

In response to the COVID-19 pandemic, many countries, including Italy, expanded vaccine administration to non-traditional settings such as pharmacies and schools [7,8,9,10]. This strategy facilitated increased vaccination coverage by leveraging local infrastructure and the public’s trust in pharmacists [11,12]. Currently, pharmacists are authorized to administer vaccines in 15 European countries, predominantly for influenza and COVID-19, with some countries extending this authorization to a broader range of vaccines, including those for pneumococcal disease, herpes zoster, meningococcal disease, and human papillomavirus (HPV) [7]. Although expanding vaccination services beyond traditional medical settings offers substantial public health benefits, it underscores the importance of accurately quantifying the risk of rare but serious adverse events, such as anaphylactic shock and fatal outcomes. Administering vaccines in non-medical environments, where immediate access to advanced medical care may be limited, makes assessing the risk of these severe reactions even more critical.

Despite the relevant research on anaphylaxis following COVID-19 vaccines [13,14,15], there is a notable lack of comprehensive, global studies that evaluate the risk of anaphylaxis associated with other vaccines in adults. The limited data available are often outdated or do not cover a broad range of vaccines, further emphasizing the need for systematic investigation.

In this study, the aim is to address this gap by conducting a systematic review and meta-analysis of the incidence of post-vaccination anaphylaxis in adults. Through examining the available evidence, in this study, the aim is to provide a clear and up-to-date overview of the risk of anaphylaxis across various vaccines. It will also estimate any differences in risk across vaccine types.

## 2. Materials and Methods

### 2.1. Protocol Registration

This systematic review and meta-analysis adhered to the Cochrane Collaboration guidelines and followed the Preferred Reporting Items for Systematic Reviews and Meta-Analyses (PRISMA) [16] and MOOSE guidelines [17] for reporting results. The study protocol was pre-established, shared among the research team, and registered in PROSPERO (registration number: CRD42024566928).

### 2.2. Literature Search Strategy

A comprehensive literature search was performed across four electronic databases of PubMed/MEDLINE, EMBASE, Scopus, and Web of Science on 8 July 2024. The search strategy was developed around three key elements of anaphylaxis, vaccines, and adults. No time limits were applied, and only articles published in English were considered. Keywords, including MeSH terms and title/abstract, were combined using the Boolean operators AND and OR. The search string used for PubMed is provided in Table S1. Search strategies for other databases were adapted based on this string to accommodate the specific syntax and indexing of each database. Additional articles were retrieved screening references of included studies or consulting experts in the field.

### 2.3. Eligibility Criteria

Inclusion and exclusion criteria were defined according to the Population, Intervention, Comparison, Outcome, and Study Design (PICOS), as recommended by the Cochrane Review. Studies involving adults aged 18 years or older who had received one or more vaccines and reporting the incidence of post-vaccination anaphylaxis as either a primary or secondary outcome were eligible. All vaccinations were included, except for COVID-19 vaccines. COVID-19 vaccine studies were excluded to avoid potential bias due to increased surveillance and reporting during the pandemic, ensuring comparability among vaccines and the reliability of the reported incidence rates. Both studies addressing general anaphylaxis and those focusing specifically on anaphylactic shock diagnosed according to well-defined criteria were considered. Only original observational studies published in English in international, peer-reviewed scientific journals were included. Excluded were non-original studies (letters to editor, book chapter, reviews, commentary), those that did not specifically assess the number or incidence of anaphylaxis, studies on COVID-19 vaccines, studies involving individuals younger than 18 years, and non-English publications. A detailed description of the inclusion criteria, following PICOS, is provided in Table S2.

### 2.4. Study Selection and Data Extraction

The study selection was performed independently by two reviewers (F.P. and A.C.D.) in the following two phases: (1) the initial screening of titles and abstracts and (2) by full-text assessment. Discrepancies were resolved through discussion or consultation with a third senior researcher (V.G.). A PRISMA flow diagram was used to outline the study selection process, including the number of studies identified, screened, excluded, and included in the final analysis, with reasons for exclusions documented. Data were extracted using a predefined spreadsheet in Excel (Microsoft Excel^®^ for Microsoft 365 MSO, Redmond, WA, USA, 2019), pre-tested on five randomly selected studies.

Extracted data included the following:

1. Study characteristics: author, publication year, country, study period, and description of data collection method.
2. Characteristics of vaccinated participants: study population, sample size (total number of participants and number of vaccinated people or administered doses), mean age, and gender distribution.
3. Characteristics of participants with anaphylaxis: allergic history and symptoms
4. Vaccine details: vaccine type and number of doses.
5. Anaphylaxis details: number of anaphylaxis cases, diagnostic criteria, severity classification, time to onset (defined as the time between vaccination and onset of anaphylaxis), treatment, and outcome.

Data extraction was performed in duplicate, and discrepancies were resolved by discussion.

### 2.5. Risk of Bias Assessment

The risk of bias in the included studies was evaluated using the Joanna Briggs Institute (JBI) Critical Appraisal Checklist for observational studies [18]. The JBI’s tools offer specific checklists for various study types, including analytical cross-sectional studies, case-control studies, case reports, case series, cohort studies, and others. We selected the appropriate checklist based on the specific study design.

This tool assesses key methodological domains such as participant selection, measurement of exposure and outcomes, confounding factors, and statistical analysis.

Each checklist provides the following answer options for each criterion: Yes, No, Unclear, or Not Applicable. Each item on the checklist was scored with 1 point if the answer was “yes,” and 0 points if the answer was “no” or “unclear.” The total number of “yes” responses was then summed and divided by the total number of questions for each study, providing a proportional score to reflect the study’s risk of bias. Studies were considered for inclusion if they achieved a “Yes” score exceeding 50% of the checklist questions, establishing this threshold as a cut-off to identify high-risk of bias studies and to ensure a reasonable standard of quality and bias reduction. Each study was classified as having a low or medium risk of bias based on the cumulative assessment of these domains. Studies scoring ≥75% were classified as low risk while studies scoring between 50% and 74% were categorized as medium risk.

### 2.6. Data Synthesis

Following PRISMA 2020 guidelines, the selection process was documented with a flow diagram showing the number of references excluded at each step. Reasons for study exclusion after full-text assessment were detailed. Data synthesis was organized into tables to provide an overview of study characteristics and findings. The collected data were synthesized uniformly and consistently. To ensure comparability across studies, we harmonized the severity classifications of anaphylactic reactions. Reactions classified as mild or moderate were grouped as Level 3, corresponding to low diagnostic certainty with involvement of a single organ system. Reactions classified as severe were grouped as Level 1 or 2, indicating high diagnostic certainty or involvement of multiple organ systems. This approach allowed for a standardized assessment of severity, facilitating meaningful comparisons between studies that utilized the Brighton Collaboration Anaphylaxis Case Definition [19] and those employing clinical severity classifications.

### 2.7. Statistical Analysis

A meta-analysis was performed to pool data and estimate the event rate of anaphylactic shock, applying a logit transformation with 95% confidence intervals (CIs) due to the low incidence of the outcome [20]. The meta-analysis included studies that reported the incidence of anaphylactic shock in adult populations, with all samples exclusively comprising individuals aged 18 and above. Both fixed-effect and random-effect models were employed to account for potential variability across studies. For ease of clinical interpretation, the pooled logit values were back-transformed into event rates using the following formula:

$$Event\;Rate=\frac{e^{logit(pooled)}}{1+e^{logit(pooled)}}$$

Heterogeneity among the included studies was assessed using the I^2^ statistic. Heterogeneity was classified as follows: high (I^2^ ≥ 75%), moderate (50% ≤ I^2^ < 75%), low (25% ≤ I^2^ < 50%), or negligible (I^2^ < 25%). Publication bias was evaluated through visual inspection of funnel plots and Egger’s test, with a *p*-value of <0.10 indicating the presence of bias. In cases where publication bias was detected, the trim-and-fill method was applied to adjust for potentially missing studies. All statistical analyses were conducted using STATA version 18 and Prometa3^®^ software (Internovi, Cesena, Italy).

### 2.8. Sensitivity Analyses

Sensitivity analyses were conducted based on vaccine type to determine whether outcome variations could be attributed to this factor. Only subgroups with at least three studies per vaccine type were included in the analysis. Sensitivity analyses were also performed to evaluate the robustness of the results, enhancing the reliability and validity of the review findings. Moreover, a sensitivity analysis was performed on studies involving populations considered vulnerable due to chronic diseases or a history of allergies, to better evaluate the risk of anaphylactic shock in this group.

## 3. Results

### 3.1. Literature Search

**Figure 1:**
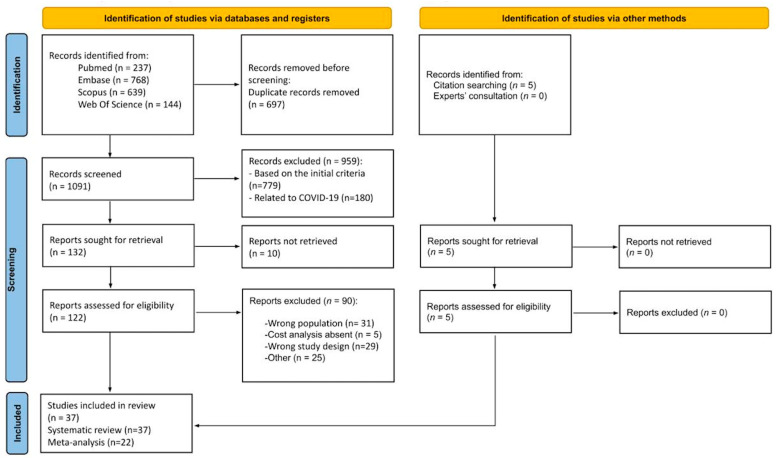
PRISMA flow diagram of study search, screen, assessment, and extraction.

A total of 1788 records were identified by searching PubMed/MEDLINE (n = 237), Scopus (n = 639), Embase (n = 768), and Web of Science (n = 144). Five additional articles [21,22,23,24,25] were included based on reference screening and expert consultation. After the preliminary exclusion of duplicates (n = 697), a total of 1091 records were screened based on title and abstract. Based on the initial screening, 779 records were removed based on the following criteria: (1) wrong population, (2) inappropriate study design, (3) wrong outcomes, (4) wrong publication type, or (5) language limitations. Additionally, 180 studies related to COVID-19 were excluded. The initial screening process yielded 132 articles for further assessment. Ten reports [26,27,28,29,30,31,32,33,34,35] were not retrieved, and after further eligibility checks, thirty-seven studies [21,22,23,24,25,36,37,38,39,40,41,42,43,44,45,46,47,48,49,50,51,52,53,54,55,56,57,58,59,60,61,62,63,64,65,66,67] were finally included in the review. However, 22 [22,23,24,36,39,40,42,43,45,47,48,49,50,54,56,57,58,60,61,62,66,67] were included in the meta-analysis because the remaining studies did not specifically report the number of anaphylactic shock cases in adults [25,41,42,55,59,64,65] or did not clearly define the adult sample [37,38,41,44,46,51,52,53,65]. The study selection process is visually represented in the PRISMA flow diagram (Figure 1), detailing the number of records identified, screened, excluded, and included in the review.

### 3.2. Descriptive Characteristics of Included Studies

**Figure 2:**
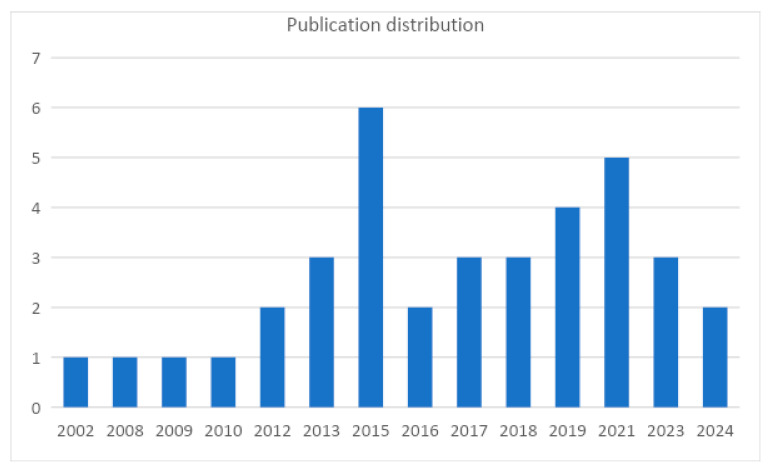
Annual publication distribution of included studies.

Figure 2 represents the annual distribution of selected publications from 2002 [57] to 2024 [56]. The frequency of publications varied year by year, with a relevant increase in publications in recent years. A primary peak was noted in 2015, with six publications [21,25,50,53,55,59].

**Figure 3:**
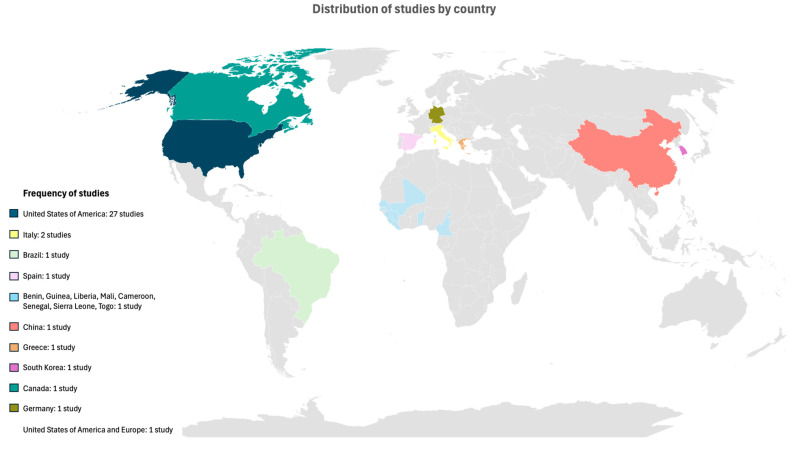
Geographical distribution of studies conducted by country.

The included studies were carried out across various geographical regions, including America (n = 28) [21,22,25,36,40,41,42,43,44,45,47,48,49,50,51,52,53,54,55,56,57,59,60,61,63,64,65,67], Europe (n = 5) [24,37,39,58,62], Asia (n = 2) [46,66], and Africa (n = 1) [38] and one [23] involving 105 sites between North America and Europe (Figure 3).

A strong reliance on electronic health data and reporting systems for monitoring vaccine safety was observed, with the most frequent method being the Vaccine Adverse Event Reporting System (VAERS) (n = 11) [25,44,49,51,52,53,55,59,63,64,65].

The sample size referred to the total number of participants in the studies, ranging from 1350 [67] to 1,594,340,278 [38]. The mean number of participants across these studies is approximately 178,537,451. The age range across all studies spanned from 13 [24] to 76.5 years [62] while 23 studies did not provide any information about participant age [25,37,38,39,40,43,44,46,49,50,51,52,53,54,55,56,57,58,59,63,64,65,67]. Gender distribution was provided in 11 studies [25,44,49,50,51,52,53,59,63,64,65], with percentages referring to the proportion of female participants (mean of female participants 56.86%). Notably, in two studies [21,47], the study population was entirely female; in the first case, two females experienced delayed anaphylactic reactions to the influenza vaccine, and in the second, the study focused on pregnant women who received the Tdap vaccine (Table 1).

Out of 37 studies, 30 [21,22,25,36,37,38,39,40,41,43,44,46,49,50,51,52,53,54,55,56,57,58,59,60,61,62,63,64,65,66] were classified as general population studies, while 7 [24,42,45,47,48,58,67] were categorized as subgroup studies focusing on individuals with chronic conditions or a history of allergies or other significant health statuses. These subgroup studies included specific populations such as adult patients with chronic kidney disease on regular dialysis therapy [42], HIV-infected adults [45], pregnant women [47], patients with immune-mediated inflammatory diseases [48], adults with a history of vaccination-associated anaphylaxis [24], dialysis patients and staff at a single hemodialysis unit [58], and adults who had experienced an adverse event following immunization (AEFI) and re-immunized [67].

Dose-specific information was not largely available (n = 18); two studies [41,48] provided data both on the first and second dose, while eight studies [21,23,49,56,58,61,66,67] focused on administered single doses, one study [22] analyzed third doses, and one [60] described booster doses. Only six studies [43,45,50,54,55,63] provided data on multiple doses (first, second, third, and fourth). One study [46] analyzed a vaccination cycle (series) of 4vHPV vaccine.

The analysis of vaccine types per study showed a predominance of influenza vaccines (n = 15) [21,24,25,37,39,43,45,50,52,53,56,58,62,64,65], followed by pneumococcal vaccines (n = 8) [43,44,45,50,57,61,66,67], Td/Tdap, (n = 7) [24,43,45,47,50,60,67], HZV (n = 6) [23,43,48,50,51,54], hepatitis B (n = 5) [24,36,43,45,67], HPV (n = 4) [40,46,50,67], and yellow fever (n = 3) [38,42,49]. Hepatitis A [24,50], Japanese encephalitis [55,63], and meningococcal [41,50] are represented in two studies each (n = 2). Varicella [50], rabies [50], rotavirus [67], and tick-borne encephalitis [24] are each the focus of one study (n = 1).

### 3.3. Anaphylaxis Across Vaccines

**Figure 4:**
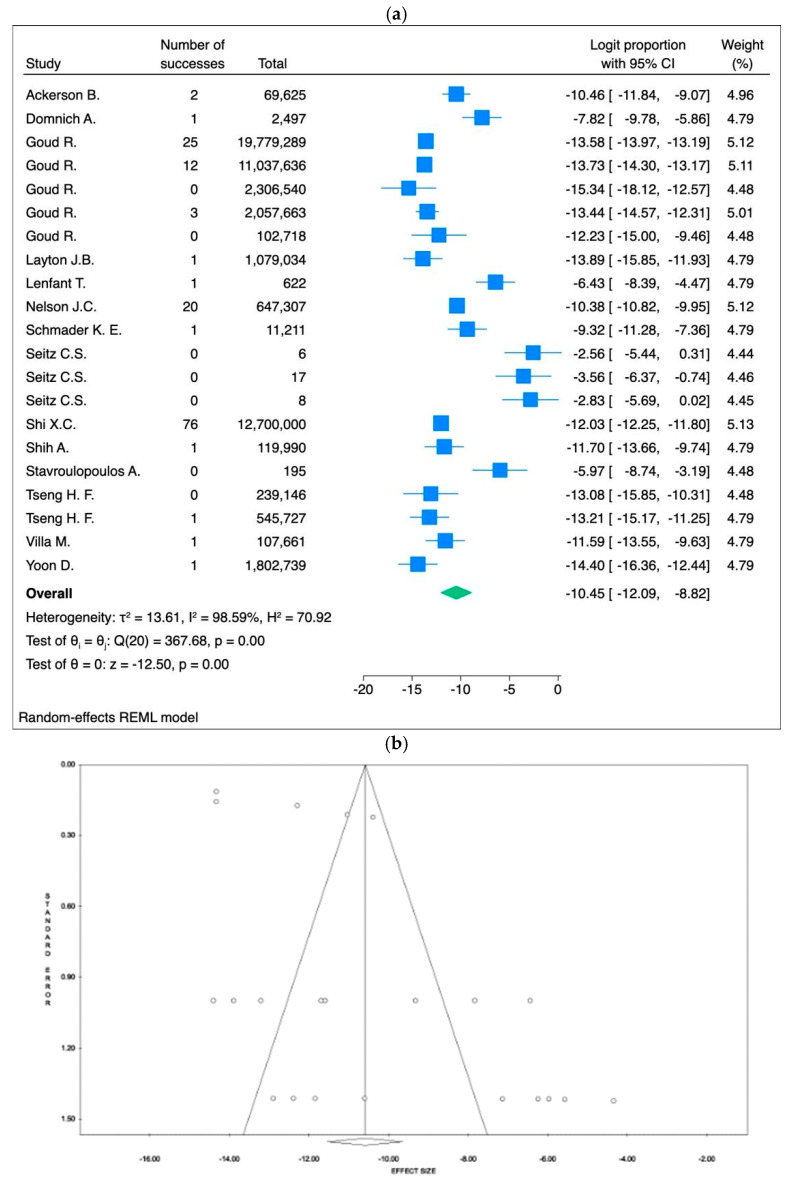
(**a**) Forest plot and (**b**) funnel plot of the random-effects model assessing the logit proportion among all vaccine types. In the forest plot, the effect sizes of individual studies are represented in blue, while the overall effect size is shown in green.

Regarding the overall number of anaphylaxis per type of vaccine (Table 2, Figure 4), of the 262 recorded cases, 153 were linked to the influenza vaccine [21,25,37,39,43,50,52,53,56,62,64,65], followed by yellow fever (n = 29) [38,49], pneumococcal (22 cases) [43,44,50,57,61,66], HZV (n = 38) [23,43,48,50,51,54], and a mixed group (i.e., TIV, Tdap, herpes zoster, n = 15) [43,45,47,50,60,67]. Fewer reactions were associated with MMR (n = 13) [59], hepB-alum [36], and HPV [46] (two each), and menB [41], hepatitis A [50], and Japanese encephalitis [63] (one each). Nine studies reported zero cases of anaphylaxis [22,24,40,42,45,55,58,60,67].

Table 2 presents an overall analysis of vaccine types, revealing the incidence of anaphylaxis across the selected studies. It also highlights the anaphylaxis rate for each type of vaccine per 100,000 participants. Influenza vaccines accounted for the highest absolute number of anaphylaxis cases (n = 153). However, when considering the sample size, the rate of anaphylaxis for influenza vaccines was at 0.4 per 100,000 participants. The yellow fever vaccine, instead, exhibited the highest rate of anaphylaxis at 2.1 per 100,000 participants, indicating a higher risk in relative terms. Td/Tdap and pneumococcal vaccines, although widely distributed, showed low rates of anaphylaxis: 0.1 per 100,000 participants for Td/Tdap and 0.9 per 100,000 participants for pneumococcal vaccines.

The time to onset of anaphylaxis varied from a few minutes [41] to 14 days [38]. The fastest onset (<15 min) was observed in seven studies [23,25,49,51,61,64,65]. Some studies included in the review (n = 5) [50,51,52,53,59] utilized the Brighton Collaboration Anaphylaxis Case Definition to assess and classify anaphylactic reactions following vaccination. Other studies (n = 7) [21,38,43,48,60,63,64] used clinical severity classifications (i.e., mild, moderate, severe), with most cases being classified as moderate (Level 3) to severe (Level 1–2), requiring immediate medical intervention (Table 1).

In terms of treatment, epinephrine was commonly administered (n = 8) [23,25,43,50,59,63,64,65], alongside other supportive treatments such as antihistamines and corticosteroids (n = 9) [23,25,39,43,48,50,63,64,65], which are standard for managing anaphylactic reactions. All anaphylactic reactions led to patient recovery, except in two cases. Specifically, one case required hospitalization [50], while another case resulted in a fatal outcome [38].

### 3.4. Risk of Bias

Overall, out of the 37 selected articles, 23 [21,22,23,25,36,37,38,40,41,42,43,46,47,51,56,57,58,59,61,62,63,64,65] scored ≥75% and were categorized as low risk, while 14 [24,39,44,45,48,49,50,52,53,54,55,60,66,67] scored between 50% and 74%, classifying them as middle risk. No articles scored below 50%, meaning none of the studies were categorized as high risk. Breaking this down by study type, eight [24,36,37,38,41,63,64,67] studies were categorized as case-control, and all of them are classified as low risk. A total of 15 studies were categorized as analytical cross-sectional, of which 9 [21,25,40,42,46,51,56,59,65] were low risk and the remaining 6 [39,44,52,53,54,55] were middle risk. Additionally, 13 studies were categorized as cohort studies, with 7 [22,43,47,57,58,61,62] classified as low risk and 6 [45,47,49,50,60,62] as middle risk. Lastly, one study [23] was categorized as an RCT, and it was classified as low risk (see Table S2).

### 3.5. Meta-Analysis

The meta-analysis, based on 22 studies [22,23,24,36,39,40,42,43,45,47,48,49,50,54,56,57,58,60,61,62,66,67] and including 206,855,261 participants, revealed a low incidence of anaphylactic shock across the various vaccines assessed. The fixed-effects model produced a logit proportion of −12.07 (95% CI: −12.23 to −11.91, *p* < 0.001), corresponding to an event rate of approximately 0.57 events per 100,000 subjects (95% CI: 0.49 to 0.68). Despite this, there was significant heterogeneity (I^2^ = 94.56%, *p* < 0.001).

The random-effects model yielded a logit proportion of −10.45 (95% CI: −12.09 to −8.82, *p* < 0.001), corresponding to an event rate of 2.91 events per 100,000 subjects (95% CI: 0.56 to 14.73). Potential publication bias was detected through visual inspection of the funnel plot and confirmed by Egger’s regression test (intercept = 3.95, *p* = 0.010).

The results are presented in Figure 4a (forest plot) and Figure 4b (funnel plot), with additional forest and funnel plots for the fixed-effect models, included in Figure S1a,b.

### 3.6. Sensitivity Analysis

Sensitivity analysis by vaccine type revealed variations in the event rate across different vaccines.

**Figure 5:**
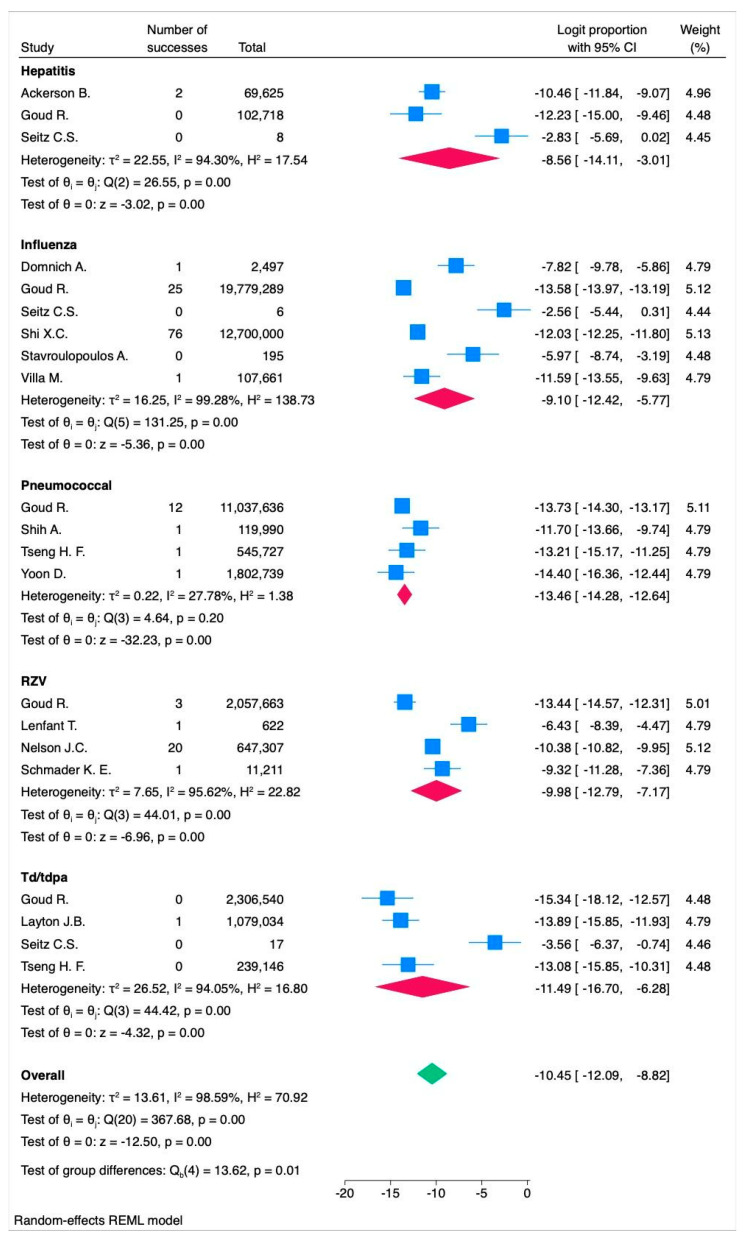
Forest plot of the random-effects model assessing the logit proportion among each vaccine type. RZV = recombinant zoster vaccine. Td/Tdap = tetanus and diphtheria or tetanus toxoid, reduced diphtheria toxoid, acellular pertussis. In the forest plot, the effect sizes of individual studies are represented in blue, the overall effect size for each vaccine type is shown in red, and the overall effect size for all vaccine types combined is displayed in green.

**Figure 6:**
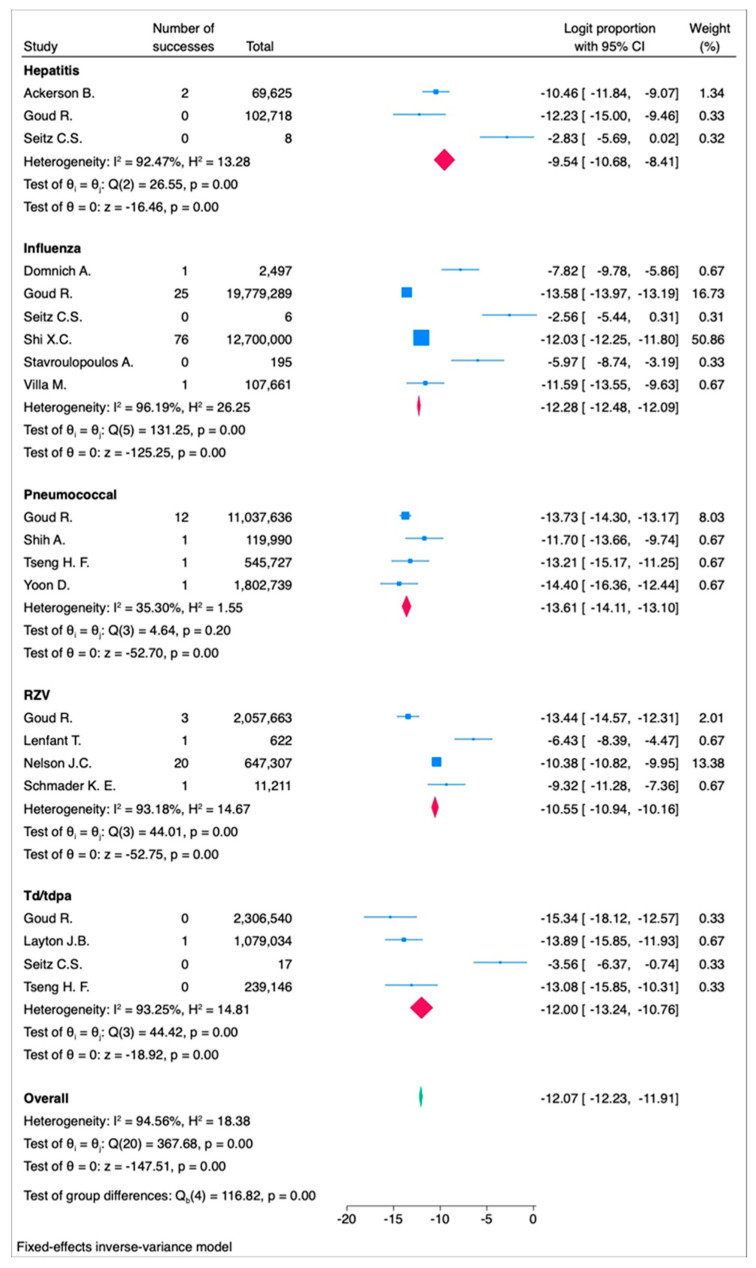
Forest plot of the fixed-effects model assessing the logit proportion among each vaccine type. RZV = recombinant zoster vaccine. Td/Tdap = tetanus and diphtheria or tetanus toxoid, reduced diphtheria toxoid, acellular pertussis. In the forest plot, the effect sizes of individual studies are represented in blue, the overall effect size for each vaccine type is shown in red, and the overall effect size for all vaccine types combined is displayed in green.

For the three studies [24,36,43] on hepatitis vaccines, the random-effects model yielded a logit proportion of −8.56 (95% CI: −14.11 to −3.01, *p* < 0.001) with high heterogeneity (I^2^ = 94.30%, *p* < 0.001), corresponding to 19.05 events per 100,000 subjects (95% CI: 0.08 to 4733.35). The fixed-effects model showed a logit proportion of −9.54 (95% CI: −10.68 to −8.41, *p* < 0.001) with high heterogeneity (I^2^ = 92.47%, *p* < 0.001), corresponding to 7.2 events per 100,000 subjects (95% CI: 2.3 to 22.15) (Figure 5 and Figure 6).

For the four studies [23,43,48,54] on RZV vaccines, the random-effects model indicated a logit proportion of −9.98 (95% CI: −12.79 to −7.17, *p* < 0.001) with high heterogeneity (I^2^ = 95.62%, *p* < 0.001), corresponding to 4.62 events per 100,000 subjects (95% CI: 0.28 to 77). The fixed-effects model showed a logit proportion of −10.55 (95% CI: −10.94 to −10.16, *p* < 0.001) with high heterogeneity (I^2^ = 93.18%, *p* < 0.001), corresponding to 2.62 events per 100,000 subjects (95% CI: 1.77 to 3.87).

For the six studies [24,39,43,56,58,62] on influenza vaccines, the random-effects model showed a logit proportion of −9.10 (95% CI: −12.42 to −5.77, *p* < 0.001) with high heterogeneity (I^2^ = 99.28%, *p* < 0.001), corresponding to 11.2 events per 100,000 subjects (95% CI: 0.4 to 310). The fixed-effects model indicated a logit proportion of −12.28 (95% CI: −12.48 to −12.09, *p* < 0.001) with high heterogeneity (I^2^ = 96.19%, *p* < 0.001), corresponding to 0.46 events per 100,000 subjects (95% CI: 0.38 to 0.56).

For the four studies [24,43,47,60] on Td/Tdap vaccines, the random-effects model yielded a logit proportion of −11.49 (95% CI: −16.70 to −6.28, *p* < 0.001) with high heterogeneity (I^2^ = 94.05%, *p* < 0.001), corresponding to 1.02 events per 100,000 subjects (95% CI: 0.005 to 187). The fixed-effects model showed a logit proportion of −12.00 (95% CI: −13.24 to −10.76, *p* < 0.001) with high heterogeneity (I^2^ = 93.25%, *p* < 0.001), corresponding to 0.614 events per 100,000 subjects (95% CI: 0.177 to 2.09).

For the four studies [43,57,61,66] on pneumococcal vaccines, the random-effects model yielded a logit proportion of −13.46 (95% CI: −14.28 to −12.64, *p* < 0.001) with high heterogeneity (I^2^ = 27.78%, *p*< 0.20), corresponding to 0.122 events per 100,000 subjects (95% CI: 0.0817 to 0.203). The fixed-effects model showed a logit proportion of −13.61 (95% CI: −14.11 to −13.10, *p* < 0.001) with high heterogeneity (I^2^ = 35.30%, *p*< 0.20), corresponding to 0.122 events per 100,000 subjects (95% CI: 0.0817 to 0.203).

**Figure 7:**
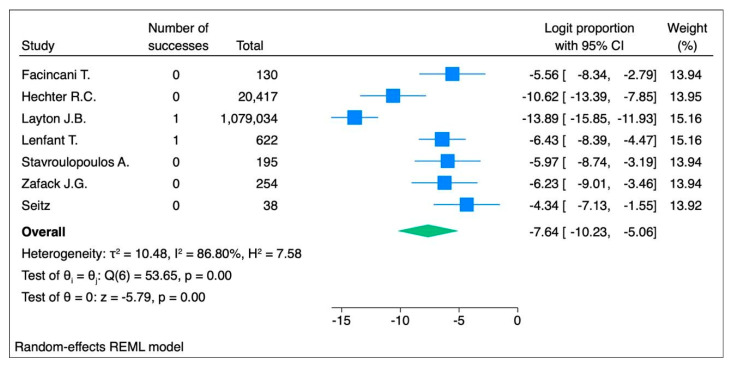
Forest plot of the random-effects model assessing the logit proportion among vulnerable populations (individuals with pre-existing allergies or chronic conditions). In the forest plot, the effect sizes of individual studies are represented in blue, while the overall effect size is shown in green.

For the seven studies [24,42,45,47,48,58,67] on the vulnerable populations (due to chronic conditions or a history of allergies), the random-effects model yielded a logit proportion of −7.64 [95% CI: −10.23, −5.06, *p* < 0.001] with high heterogeneity (I^2^ = 86.80%, *p* < 0.001), corresponding to 48 events per 100,000 subjects (95% CI: 4 to 631) (Figure 7). The fixed-effects model showed a logit proportion of −8.16 (95% CI: −9.09, −7.24, *p* < 0.001) with high heterogeneity (I^2^ = 88.82%, *p* < 0.001), corresponding to 29 events per 100,000 subjects (95% CI: 11 to 72) (Figure S2).

## 4. Discussion

### 4.1. Summary of Results

In this systematic review and meta-analysis, the incidence of anaphylaxis was assessed following non-COVID-19 vaccinations in adults, including data from 37 studies with a total sample size of 206,855,261 participants across North America, Europe, Asia, and Africa. The fixed-effects model estimated a pooled event rate of 0.57 per 100,000 individuals (95% CI: 0.49–0.68), while the random-effects model reported a higher rate of 2.91 per 100,000 (95% CI: 0.56–14.73), with significant heterogeneity (I^2^ = 94.56%, *p* < 0.001). Publication bias was suggested by the funnel plot and confirmed by Egger’s regression test (intercept = 3.95, *p* = 0.010). The meta-analysis revealed significant variability in anaphylactic event rates across vaccine types. Influenza vaccines exhibited the highest event rate, with up to 11.2 events per 100,000 individuals (95% CI: 0.4–310) under the random-effects model, reflecting substantial heterogeneity (I^2^ = 99.28%). In contrast, pneumococcal vaccines (PCV13 and PPSV23) showed the lowest event rates, with an estimated 0.122 events per 100,000 individuals (95% CI: 0.0817–0.203) consistently reported under both fixed- and random-effects models, demonstrating minimal variability. Gender differences were also noted, particularly for the MMR vaccine, where 77% of the reported cases were female [59,68,69]. Despite the low incidence of severe outcomes, the timely administration of appropriate medical interventions, such as epinephrine, generally resulted in positive outcomes. The time to onset of anaphylaxis varied widely, ranging from a few minutes to as long as 14 days post-vaccination, underscoring the importance of both immediate and extended post-vaccination surveillance to effectively manage delayed reactions.

### 4.2. Interpretation of the Results

Anaphylaxis following vaccination is rare, and estimated occurrence varies with the surveillance systems used to obtain data [70]. In the United Kingdom, national active surveillance identified a rate of 12 cases per 100,000 doses distributed after a single-component measles vaccine among children aged less than 16 years [71]. Reporting from selected healthcare organizations in the United States found an overall rate of anaphylaxis after vaccination of 1.3 cases per million doses administered to both children and adults [48]. In this review, the data reflected this rarity and were consistent with analyses of other passive reporting systems describing the frequency of anaphylaxis after vaccination [71,72]. However, no recent studies have systematically and quantitatively analyzed the incidence of anaphylaxis across a wide range of vaccines without geographical limitations. With the general COVID-19 vaccine rollout in December 2020, there were immediate reports of anaphylaxis following mRNA COVID-19 vaccination at an incidence of 7.91 cases per million doses [13,14]. Therefore, our study addressed this gap by including a broader spectrum of vaccines and global data, providing a more comprehensive assessment of the scientific evidence of vaccine-related anaphylaxis across different populations and geographical settings.

The frequency of publications on anaphylaxis following vaccination has shown variation over the years, with a notable peak in 2015, during which six studies [21,25,50,53,55,59] were published. This increase in research activity can be linked to heightened concerns about vaccine safety, particularly in the context of rising vaccine hesitancy from 2009 to 2014. During this period, vaccine hesitancy expanded globally, largely driven by misinformation and public fear, particularly surrounding unfounded claims linking vaccines to severe adverse outcomes. The situation was further compounded by events such as the suspension of the Fluad vaccine [73] batches in Italy in 2014, which raised public alarm despite subsequent investigations showing no causal link between the vaccine and reported deaths [74]. The spike in 2015 publications likely reflects the scientific community’s response to these concerns, aiming to provide robust data on rare but serious adverse events like anaphylaxis and to counteract the growing skepticism surrounding vaccine safety with evidence-based research [75]. By improving the precision and comprehensiveness of vaccine safety assessments, particularly for non-COVID vaccines, public health authorities can better address concerns and promote informed decision-making among the public [76].

Some findings in this analysis are consistent with previous observations. The predominance of the female sex in older age groups has been observed in previous analyses [48,70,77]. Most included studies (n = 15) [21,23,25,39,41,43,49,50,51,52,54,59,61,64,65] noted symptoms within the typical window for IgE-mediated responses, with onset most commonly within minutes to a few hours post-vaccination [48,78]. Notably, we documented a rare case where the onset of anaphylaxis was delayed, occurring up to 14 days after vaccination [34]. Delayed-onset reactions, while rare, have been reported in response to vaccines such as rabies and Japanese encephalitis [79]. This extended timeframe suggested that delayed reactions, although infrequent, may occur and require monitoring beyond the immediate post-vaccination period. However, it is possible that these reports might reflect diagnostic errors or confounding factors, such as unrelated allergic events occurring after vaccination. Improved documentation, clearer diagnostic criteria, and awareness of potential biases are crucial to avoid misattribution. In this perspective, immunization information systems and other digital technologies can be helpful in supporting vaccination program and adverse events surveillance. Monitoring patients beyond the immediate post-vaccination period, especially those with complex medical histories, can help ensure that delayed reactions are appropriately managed and correctly classified. Symptoms ranged from milder signs like urticaria, rash, pruritus, and flushing to more severe cases involving respiratory distress, throat tightness, angioedema, and vomiting. The rapid progression of symptoms in severe cases makes timely intervention critical. These observations underscore the current recommendations that any provider administering vaccines should have emergency protocols and supplies on hand, including epinephrine, should a patient develop anaphylaxis. Even when vaccinations are administered in non-traditional settings, such as pharmacies, it is essential that necessary medical supplies and trained personnel are available to manage potential anaphylactic reactions effectively. All staff should be proficient in recognizing the early signs of anaphylaxis and be prepared to initiate emergency care immediately, as any delays in response can lead to poor outcomes. Despite the sometimes dramatic presentation of symptoms, almost all fully recover [48,71].

While these reactions are more frequently observed in individuals with atopic conditions, they can also occur in those without a prior history of allergic disease [70]. The results of this meta-analysis provide critical insights into the low overall incidence of anaphylactic events associated with vaccines, while also highlighting substantial heterogeneity across vaccine types and populations. The significantly higher event rate observed for influenza vaccines compared to other vaccine types, particularly under the random-effects model, suggests potential variability in study populations, reporting standards, or underlying risk factors. Conversely, the consistently low rates for pneumococcal vaccines indicate a reassuring safety profile with minimal variability across studies. The wide confidence intervals for the random-effects estimates, especially for influenza and hepatitis vaccines, emphasize the influence of outlier studies and heterogeneity in the pooled data. These findings underscore the need for tailored risk communication, particularly for vaccines with higher reported rates, to reassure the public while addressing the specific needs of high-risk groups, such as individuals with chronic conditions or a history of allergies. Moreover, they underscore the importance of ensuring that healthcare providers are well-prepared to respond to vaccine-related emergencies, as well as maintaining robust monitoring systems to identify, manage, and minimize such events [80].

A major limitation in interpreting the results of this analysis is the significant heterogeneity in the classification and diagnosis of anaphylaxis across the included studies. While some studies (n = 5) employed standardized diagnostic criteria, such as the Brighton Collaboration Anaphylaxis Case Definition, others relied on varying clinical definitions, hospital codes, or patient-reported symptoms. This inconsistency in defining anaphylaxis introduces substantial variability in the reported outcomes and complicates the synthesis of data across studies. Consequently, the ability to draw definitive conclusions about the true incidence is impaired. To improve the reliability of future research, there is a pressing need for the adoption of standardized, rigorous criteria for the diagnosis and classification of anaphylaxis, particularly in vaccine safety surveillance studies. This will enhance the accuracy of pooled analyses and facilitate more robust conclusions regarding vaccine-associated risks. Future studies should aim to integrate real-time electronic health records (EHR) and automated surveillance systems that could capture detailed clinical data and improve the timeliness and accuracy of anaphylaxis diagnosis.

Furthermore, examining vaccine-specific differences in anaphylaxis risk is important in order to make some distinctions emerge between absolute case numbers and relative rates of occurrence. For example, the influenza vaccine had the highest absolute number of anaphylaxis cases, reflecting its widespread use in the population. However, its anaphylaxis rate was relatively low, in contrast with higher rates of anaphylaxis observed for vaccines targeting smaller populations (yellow fever vaccine). Rates normalized by population size can help identify vaccines with higher relative risks and guide tailored safety measures.

### 4.3. Future Directions and Implications for Public Health

In this study, several critical areas were underlined where future research is needed to better understand and mitigate the risk of post-vaccination anaphylaxis in adults. First, there is a clear need for the more comprehensive reporting of adverse events in future vaccine studies. Standardizing the collection of data on vaccine dose, time to onset of reactions, and detailed participant demographics will not only allow for more accurate meta-analyses but also enhance the long-term monitoring of vaccine safety over time and across populations. Also, the findings highlight the importance of interpreting anaphylaxis rates in the context of the population size, balancing the absolute number of events with the relative risk based on the vaccine’s reach. This dual perspective allows for more informed decision-making in vaccine safety and monitoring strategies.

Additionally, while COVID-19 vaccines have been subjected to extensive safety reviews, comparable systematic reviews for other adult vaccines are lacking, particularly for vaccines like influenza and pneumococcal vaccines. These vaccines are administered to millions of adults annually, including older individuals and those with chronic conditions [81], so comprehensive safety reviews of these vaccines are essential to provide an updated risk profile [82,83]. This is particularly critical in light of demographic shifts, including aging populations and evolving comorbidities [84], which may alter the risk of severe adverse reactions such as anaphylaxis.

The public health implications of these findings are substantial. Improving safety assessments and post-vaccination monitoring protocols will not only help in identifying and preventing severe adverse reactions like anaphylaxis but also bolster public confidence in vaccination programs. With anti-vaccine sentiment remaining a challenge in many parts of the world, even among healthcare personnel [85], transparent and rigorous safety monitoring is crucial to maintaining high vaccination rates and preventing outbreaks of vaccine-preventable diseases [80].

Moreover, special attention must be given to non-hospital settings, particularly for vulnerable patients, as even though the risk of anaphylaxis is very low, it cannot be overlooked. Ensuring that such environments, including pharmacies and community centers, are adequately prepared to monitor, identify, and manage rare but severe adverse events is crucial—especially in the wake of the COVID-19 pandemic, which has expanded the role of these non-medical settings in vaccine administration. This highlights the importance of training non-medical personnel in recognizing early signs of allergic reactions and ensuring that emergency interventions, such as the administration of epinephrine, are readily available. In this perspective, pharmacists are among the non-medical personnel the most frequently involved in vaccine delivery. Therefore, training pharmacists on key vaccinology topics is of paramount importance. According to recent research, pharmacists can play a pivotal role in improving vaccination coverage, particularly for adult and high-risk populations [86]. Training programs should be tailored to improve pharmacists’ understanding of immunization schedules, vaccine safety profiles, and management of adverse events, enabling them to offer accurate information and reassurance to the public [15]. Expanding these monitoring capabilities in non-traditional vaccination settings will be vital in safeguarding public health, especially as vaccine accessibility broadens to meet global immunization goals.

### 4.4. Strengths and Limitations

In this systematic review and meta-analysis, a comprehensive and up-to-date evaluation is provided of the incidence of anaphylaxis following vaccination in adults. One of the key strengths of this study is its large-scale inclusion of 37 studies, representing a total sample size of over 206 million participants across multiple geographic regions. This extensive data set enhances the generalizability of the findings and provides valuable insights into the risk of anaphylaxis for various vaccines, including influenza, yellow fever, and pneumococcal vaccines. The use of both fixed-effects and random-effects models allows for robust statistical analysis, taking into account the variability across studies and providing a range of estimates for the event rates of anaphylaxis. Additionally, in this study, sensitivity analyses are employed to account for vaccine types and specific vulnerable populations, further improving the reliability of the results. In our meta-analysis, the limited number of anaphylaxis cases prevented the direct calculation of the event rate. To address this, the logit proportion was first estimated and then converted into the event rate. A key strength of this meta-analysis lies in the use of statistical methods tailored for rare events, while also ensuring that the results are presented clearly for ease of interpretation. This methodological approach enhances the practical applicability of the findings, making them more relevant for clinical decision-making and public health policy.

However, several limitations should be acknowledged. The significant heterogeneity observed across the studies, particularly in the classification and diagnosis of anaphylaxis, poses a challenge to the accuracy of pooled estimates. The variability in diagnostic criteria introduces uncertainty in interpreting the true incidence of anaphylaxis. Another limitation is the reliance on passive surveillance systems, which may underestimate the true incidence of adverse events due to reporting biases. Furthermore, in this study, COVID-19 vaccine studies were excluded, limiting the broader applicability of the findings to the current context of global vaccination efforts. Additionally, the exclusion of data from reports and other forms of grey literature further restricts the comprehensiveness and practical relevance of the results. Lastly, while the meta-analysis provides important insights into the rates of anaphylaxis, the small number of reported cases for some vaccines (e.g., HPV and hepatitis B) restricts the ability to draw definitive conclusions regarding their safety profiles.

## 5. Conclusions

This systematic review and meta-analysis confirm that the overall risk of anaphylactic events following vaccination is exceedingly low, reinforcing the safety of vaccines across diverse populations. These findings also highlight the importance of continued surveillance and transparent reporting of adverse events to maintain public trust in vaccination programs. While the findings support the overall safety of adult vaccines, they also highlight the need for improved post-vaccination monitoring and more comprehensive adverse event reporting. By addressing these gaps, public health authorities can better safeguard vaccine safety and promote broader acceptance of vaccination programs, ultimately enhancing the global effort to prevent infectious diseases.

## Figures and Tables

**Table 1:** Study details including data collection methods and population characteristics.

Author	Country	Study Period	Vaccine Type	Description of Data Collection Method	Study Population	Gender Distribution (Female)	Mean Age (± SD)	Allergic History	Number of Anaphylaxis Cases	Time to Onset	Symptoms	Quality Score
Ackerson B, 2023 [36]	USA	2018 (Jul 8)–2019 (Oct 31)	Hepatitis B	Electronic health records	Adults not on dialysis	49.0%	49 years (38–56)	NA	2 (HepB-alum)	NA	NA	8/10
Alguacil-Ramos A.M, 2019 [37]	Spain	2017–2018	Influenza (subunit and adjuvanted)	Vaccine information system (SIV)	Vaccinated population against influenza	NA	NA	NA	2	NA	NA	8/10
Breugelmans J.G., 2013 [38]	Africa	2007–2010	Yellow fever	Vaccine pharmacovigilance systems	All individuals vaccinated	NA	NA	NA	7	1.8 days (range 0–14 days)	Respiratory distress, edema, generalized urticaria	8/10
David J., 2015 [21]	USA	2010	Influenza	Case reports	Two adults with delayed anaphylactic reactions to the influenza vaccine	100%	56 years (42–71)	Latex, radiocontrast and amoxicillin	2	<5 h	Respiratory symptoms, erythematous hands, urticaria	7/8
Domnich A., 2023 [39]	Italy	2019/2020, 2020/2021, 2021/2022	Influenza	Medical records from the enhanced passive safety surveillance (EPSS)	All individuals vaccinated in the three seasonal influenza campaign	NA	NA	NA	1	1 h	swollen tongue, lip swelling, ling face, dyspnea, tachycardia, vomiting, hand erythema, cold sweats, tinnitus and paresthesia	5/8
Donahue J. G., 2019 [40]	USA	2015–2017	HPV	Six vaccine Safety Datalink sites	Women and men	64.0%	NA	18–26 years	0	NA	NA	6/8
Duffy J.,2017 [41]	USA	2013–2014	MenB-4C	Medical records	Students	52%	20 years (16–65)	NA	1	30 min	Swelling, throat	8/10
Facincani T., 2016 [42]	Brazil	2012–2014	Yellow fever	Questionnaire	Adults with chronic kidney disease on dialysis	36%	53.9 years (± 14.9)	NA	0	NA	NA	6/8
Goud R., 2021 [43]	USA	2015–2019	Influenza, pneumococcal, hepatitis B, Tdap, zoster	Medicare Fee For Service (FFS) claims data	Medicare Fee For Service beneficiaries	58%	NA	NA	31 (influenza); 18 (pneumococcal); 3 (zoster)	<24 h	NA	10/11
Haber P., 2016 [44]	USA	2012 2015	PCV13	Medical records from the Vaccine Adverse Event Reporting System (VAERS)	Adults aged 19–64 years old with chronic medical conditions and >64 years old	NA	NA	NA	1	NA	NA	5/8
Hechter R.C., 2019 [45]	USA	2002–2013	Influenza, PPSV23, PCV13, hepatitis B, Td, Tdap	Vaccine Safety Datalink	HIV-infected adults	10%	51 years (± 11.5)	NA	0	0–6 days	NA	8/11
Hu Y., 2021 [46]	China	2018–2020	HPV	National Adverse Event Following Immunization Surveillance System	Female and male participants, primarily aged 30–39 years	NA	NA	NA	2	<48 h	NA	6/8
Layton J.B., 2017 [47]	USA	2010–2014	Tdap	MarketScan Commercial Claims and Encounters databases	Pregnant women	100%	29.2 years (± 5.4)	NA	1	<48 h	NA	9/11
Lenfant T., 2021 [48]	USA	2018–2020	Herpes zoster vaccine	Electronic medical records	Patients with immune-mediated inflammatory diseases	67%	67 years	NA	1	NA	NA	7/11
Lindsey N.P., 2008 [49]	USA	2000–2006	Yellow fever	Vaccine Adverse Event Reporting System	Civilians who received the yellow fever vaccine	NA	NA	NA	22	15 min–24 h	Urticaria Rash Pruritus Flushingrespiratory symptoms	6/11
Marin M., 2021[22]	USA	2009–2010	MMR	Self-reporting, phone calls, electronic health records	Young adults, vaccinated with 2 doses of MMR	56%	20 years (18–28)	NA	0	NA	NA	10/11
McNeil M. M., 2015 [50]	USA	2009–2011	Influenza, Tdap/DTaP, HPV,MMR,VAR,HAV,HZV,PPSV23 meningococcal vaccine, rabies vaccine	Vaccine Safety Datalink (VSD)	Children and adults who received vaccines during the study period	NA	NA	Drug: 9 Asthma: 6 rhinitis: 5 dermatitis: 1 Other: 2 No allergies: 3	15	30 min–24 h	Respiratory, Gastrointestinal, Cutaneous, and Cardiovascular symptoms	7/11
Miller E.R., 2018 [51]	USA	2006–2015	Herpes zoster vaccine (live)	Vaccine Adverse Event Reporting System (VAERS)	Elderly population recommended for vaccination with ZVL	NA	NA	Drug: 4 Food: 2 Environmental: 1 Substance-specific: 2 Insect: 1	11 (7 ZVL only)	10 min–72 h	NA	6/8
Moro P.L., 2012 [52]	USA	2010–2010	Influenza (inactivated monovalent)	Vaccine Adverse Event Reporting System	≥65 years	NA	NA	NA	3	<24 h	NA	5/8
Moro P.L., 2015 [53]	USA	2013–2015	Influenza	Vaccine Adverse Event Reporting System	Persons vaccinated with ccIIV3	NA	NA	NA	2	NA	NA	5/8
Nelson J.C., 2023 [54]	USA	2018–2019	Herpes zoster vaccine	CDC Vaccine Safety Datalink	Adults aged ≥ 50 y old	58%	NA	NA	20	<24 h	NA	5/8
Rabe I. B., 2015 [55]	USA	2009–2012	Japanese encephalitis	Vaccine Adverse Event Reporting System	NA	48%	NA	NA	0	NA	NA	5/8
Schmader K. E., 2012 [23]	North America, Europe	2007–2010	Herpes zoster vaccine	Self-reporting via phone calls	History of varicella	62%	54.9 years (± 2.8)	NA	1	15 min	NA	11/13
Seitz C.S., 2009 [24]	Germany	2000–2007	Td, hepatitis A/B, tick-borne encephalitis, influenza	Patients referred by health care professionals	Adults with a history of vaccination-anaphylaxis	74%	48 years (13–79)	Atopic disease (10 pt)	0	NA	NA	7/10
Shi X.C., 2024 [56]	USA	2022–2023	Influenza	Medicare claims data	Medicare beneficiaries	NA	NA	NA	76	<24–48 h	NA	8/8
Shih A., 2002 [57]	USA	1999	PPV	Medicare claims data	Medicare beneficiaries	61%	NA	NA	1	NA	NA	11/11
Stavroulopoulos A., 2010 [58]	Greece	2007–2008	Influenza H1N1 (inactivated adjuvanted)	Prospective examination	Dialysis patients	NA	NA	NA	0	NA	NA	10/11
Sukumaran L., 2015 [59]	USA	2003–2013	MMR	Vaccine Adverse Event Reporting System	U.S. adults receiving MMR vaccine	NA	NA	History of allergies: 9	13	<24 h	Urticaria, difficulty breathing	8/8
Tseng H. F., 2013 [60]	USA	2006–2010	Tdap, Td	Diagnostic codes	≥65 years	NA	≥65 years	NA	0	NA	NA	8/11
Tseng H. F., 2018 [61]	USA	2011–2015	Pneumococcal vaccine	Vaccine Safety Datalink Project	≥65 years	55%	≥65 years	Allergy to eggs and intravenous contrast	1 (PVC23)	<15 min	Swollen tongue, breathing difficulty	9/11
Villa M., 2013 [62]	Italy	2006-2009	Influenza:	Hospital records	≥65 years	NA	75 years	NA	1	NA	NA	9/11
Walker W.L., 2018 [63]	USA	2012–2016	Japanese encephalitis	Vaccine Adverse Event Reporting System	General population	NA	NA	NA	1	NA	Angioedema, throat closure	9/10
Woo E. J., 2015 [25]	USA	2013–2014	Influenza	Vaccine Adverse Event Reporting System	Adults with a self-reported egg allergy or previous allergic reaction to inactivated influenza vaccine	NA	NA	Egg: 8 Drug: 3 Food: 6 Environmental: 3 Substance-specific: 2 vaccine-specific: 3 Other: 1	12	10 min 2 days 15 min < 5 min < 1 day 15 min 2–3 min 1.5 day < 1 day 1 day seconds	Cutaneous, Respiratory, Angioedema Gastrointestinal, Cardiovascular, Neurological	6/8
Woo E.J., 2017 [64]	USA	2013–2016	Influenza	Vaccine Adverse Event Reporting System	≥18 years	NA	NA	eggs and other substance	10	<10 min or <24 h	NA	8/10
Woo E. J., 2021 [65]	USA	2017–2020	Influenza	Vaccine Adverse Event Reporting System	Recipients of the influenza vaccine			History of allergies (75% patients)	4	<10 min	Respiratory difficulties, pruritus, swelling	6/8
Yoon D., 2024 [66]	South Corea	2018–2021	Pneumococcal	Korea Immunization Registry Information System and National Health Information Database	History of PPSV23 vaccination and cardiovascular, neurological, immunological events.	54%	68.26 years (5.57)	NA	1	NA	NA	8/11
Zafack J.G., 2019 [67]	Canada	1998–2016	Tdap, PCV, rotavirus, MMR, hepatitis B, HPV	Surveillance system’s electronic database	History of adverse events following immunization	59%	NA	NA	0	NA	NA	7/10

**Table 2:** Overall analysis of vaccine types.

Type of Vaccine	Number of Anaphylaxis (n. Cases)	Total Sample Size (Participants)	Studies Without Sample Size	Anaphylaxis Rate per 100,000 Participants
Influenza	153	32,589,648	5	0.4
Herpes zoster virus (HZV)	38	2,069,496	1	1.8
Yellow fever	29	1,355,847	1	2.1
Pneumococcal	24	2,488,873	1	0.9
Measles, mumps, and rubella (MMR)	0	662	1	0.0
Td/Tdap	5	3,505,164	0	0.1
Hepatitis B	2	172,351	0	1.1
Human papillomavirus (HPV)	2	200,298	1	0.9
Hepatitis A	1	Not specified	0	NA
Japanese encephalitis	1	Not specified	1	NA
MenB-4C	1	Not specified	1	NA
Tick-borne encephalitis	0	7	0	0.0
Rotavirus	0	Not specified	0	NA

## Data Availability

Not applicable.

## Supplementary Materials

The following supporting information can be downloaded at: https://www.mdpi.com/article/10.3390/vaccines13010037/s1, Figure S1: (a) Forest plot and (b) funnel plot of the fixed effect model assessing the Logit proportion among all vaccine types; Table S1: Literature search strategy (Pubmed).; Table S2: Inclusion criteria declined according to Population, Intervention, Comparison, Outcome, and Study design (PICOS) guidelines; Table S3: Risk of bias classification of studies; Figure S2. Forest plot of the fixed model assessing the Logit proportion among vulnerable populations (individuals with pre-existing allergies or chronic conditions).
