# Supplementary material for: Post-Vaccination Anaphylaxis in Adults: A Systematic Review and Meta-Analysis
Source: Vaccines (Basel). 2025 Jan 4;13(1):37. doi: 10.3390/vaccines13010037 (PMC11769139; doi:10.3390/vaccines13010037)

Supplementary materials

Table S1. Literature search strategy (Pubmed).

SET	Key words	Field	Component	Records retrieved (n.)
1	"vaccines"	[MeSH Terms]	Anaphylaxis following vaccination	
2	"immunization"		Immediate hypersensitivity reaction	
3	"vaccin*"	[tiab]	Responses to vaccination	
4	"immuniz*"			
5	"immunis*"		Vaccines licensed for human use	
6	**Sets 1-5 were combined with "OR"**			637,461
7	"anaphylaxis"	[MeSH Terms]		
8	"anaphylaxis react*"	[tiab]		
9	"anaphylactic react*"			
10	"anaphylactic shock*			
11	"anaphylactoid syndrome*"			
12	"anaphylactoid react*"			
13	"anaphylactic syndrome*"			
14	"anaphylactoid shock*"			
15	"acute systemic allergic react*"			
16	"idiopathic anaphylaxis"			
17	"systemic anaphylaxis"			
18	**Sets 7-17 were combined with "OR"**			38,401
19	**Sets 6 and 18 were combined with "AND"**			3,523
20	"incidence"	[tiab]	Study types	

21	"prevalence"			
22	"epidemiolog*"			
23	"cross sectional stud*"			
24	"cohort analy*"			
25	"longitudinal stud*"			
26	"prospective stud*"			
27	"retrospective stud*"			
28	"cohort stud*"			
29	"regist*"			
30	**Sets 20-29 were combined with "OR"**			3,450,660
31	**Sets 19 and 30 were combined with "AND"**			243
32	"adult"	**[MeSH Terms]**		
33	"middle aged"			
34	"aged"			
35	adult*	**[tiab]**		
36	"middle aged"			
37	"aged"			
38	**Sets 31-37 were combined with "OR"**			9,246,945
39	**Sets 31 and 38 were combined with "AND"**			89
40	"editorial"	**[Publication Type]**		
41	"comment"			
42	"abstract"			
43	"review"			
44	**Sets 17-22 were combined with "OR"**			4,868,109
45	**Sets 39 and 44 were combined with "NOT"**			237
25	**Set 24 was limited to 8th July 2024**			

Table S2. Inclusion criteria declined according to Population, Intervention, Comparison, Outcome, and Study design (PICOS) guidelines.

	**Inclusion criteria**
**Population (P)**	Adults aged 18 years or older who had received one or more vaccines administered for any indication.
**Intervention (I)**	Administration of any vaccine, excluding COVID-19 vaccines.
**Comparison (C)**	Not applicable.
**Outcome (O)**	Incidence of post-vaccination anaphylaxis as a primary or secondary outcome.
**Study design (S)**	Focused on anaphylactic reactions resulting from COVID-19 vaccine administration.
**Time filter**	None

Table S3. Risk of bias classification of studies

Year of Publication	Author	Study Type	YES (%)	Quality Score	Risk Classification
2023	Ackerson B.	Case-Control	80%	8/10	Low
2019	Alguacil-Ramos A.M.	Case-Control	80%	8/10	Low
2013	Breugelmans J.G.	Case-Control	80%	8/10	Low
2015	David J.	Analytical Cross-Sectional	87.5%	7/8	Low
2023	Domnich A.	Analytical Cross-Sectional	62.5%	5/8	Middle
2019	Donahue J.G.	Analytical Cross-Sectional	75%	6/8	Low
2017	Duffy J.	Case-Control	80%	8/10	Low
2016	Facincani T.	Analytical Cross-Sectional	75%	6/8	Low
2021	Goud R.	Cohort	90.9%	10/11	Low
2016	Haber P.	Analytical Cross-Sectional	62.5%	5/8	Middle
2019	Hechter R.C.	Cohort	72.72%	8/11	Middle
2021	Hu Y.	Analytical Cross-Sectional	75%	6/8	Low
2017	Layton J.B.	Cohort	81.81%	9/11	Low
2021	Lenfant T.	Cohort	63.63%	7/11	Middle
2008	Lindsey N.P.	Cohort	54.54%	6/11	Middle
2021	Marin M.	Cohort	90.9%	10/11	Low
2015	McNeil M.M.	Cohort	72.72%	7/11	Middle

2018	Miller E.R.	Analytical Cross-Sectional	75%	6/8	Low
2012	Moro P.L.	Analytical Cross-Sectional	62.5%	5/8	Middle
2015	Moro P.L.	Analytical Cross-Sectional	62.5%	5/8	Middle
2023	Nelson J.C.	Analytical Cross-Sectional	62.5%	5/8	Middle
2015	Rabe I. B.	Analytical Cross-Sectional	62.5%	5/8	Middle
2012	Schmader K.E.	RCT	84.61%	11/13	Low
2009	Seitz C.S.	Case-Control	70%	7/10	Middle
2024	Shi X.C.	Analytical Cross-Sectional	100%	8/8	Low
2002	Shih A.	Cohort	100%	11/11	Low
2010	Stavroulopoulos A.	Cohort	90.9%	10/11	Low
2015	Sukumaran L.	Analytical Cross-Sectional	100%	8/8	Low
2013	Tseng H.F.	Cohort	72.72%	8/11	Middle
2018	Tseng H.F.	Cohort	81.81%	9/11	Low
2013	Villa M.	Cohort	81.81%	9/11	Low
2018	Walker W.L.	Case-Control	90%	9/10	Low
2015	Woo E.J.	Analytical Cross-Sectional	75%	6/8	Low
2017	Woo E.J.	Case-Control	80%	8/10	Low
2021	Woo E.J.	Analytical Cross-Sectional	75%	6/8	Low
2024	Yoon D.	Cohort	72.72%	8/11	Middle
2019	Zafack J.G.	Case-Control	70%	7/10	Middle

Figure S1. a) Forest plot and b) funnel plot of the fixed effect model assessing the Logit proportion among all vaccine types.

a)

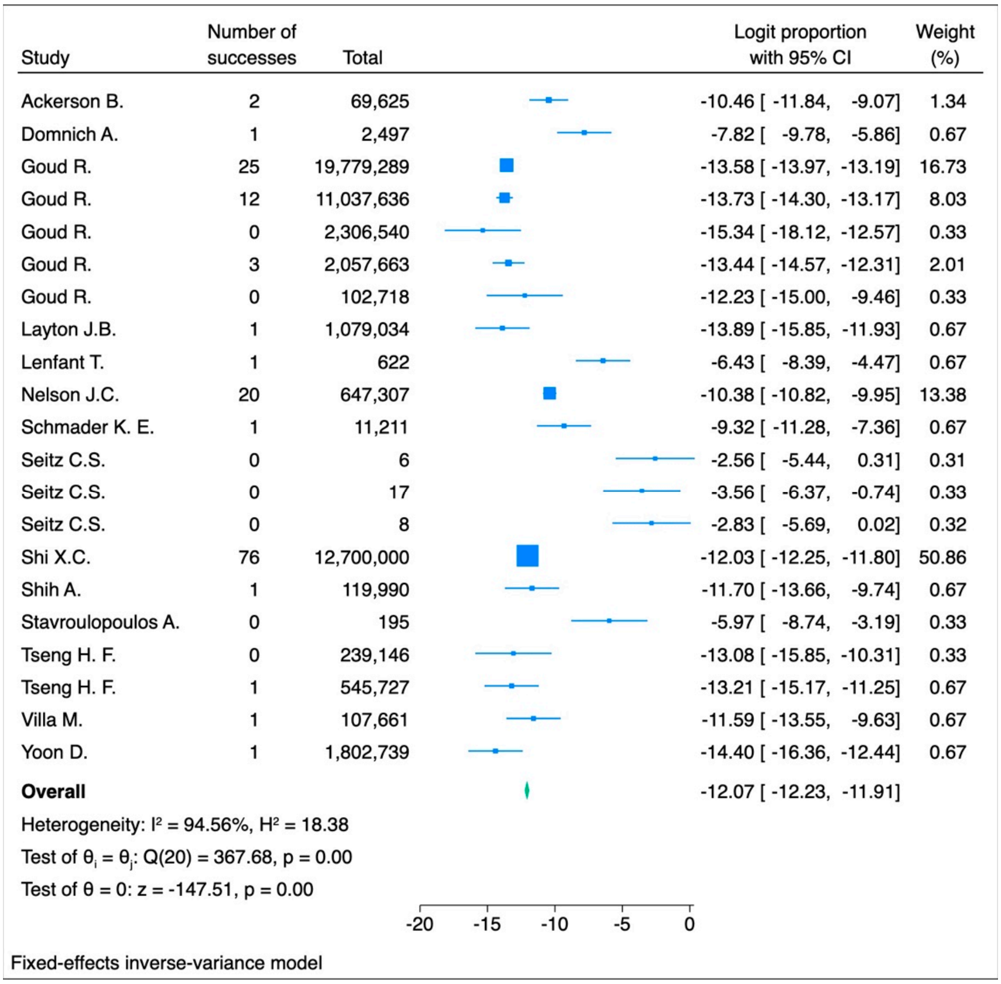

b)

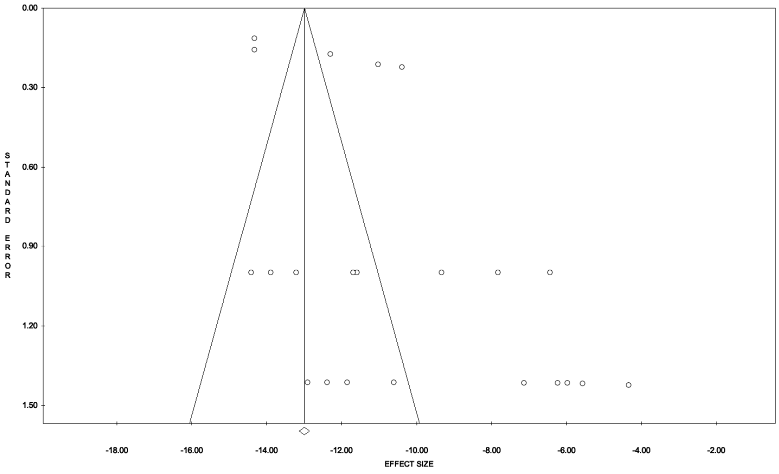

Figure S2. Forest plot of the fixed model assessing the Logit proportion among vulnerable populations (individuals with pre-existing allergies or chronic conditions).

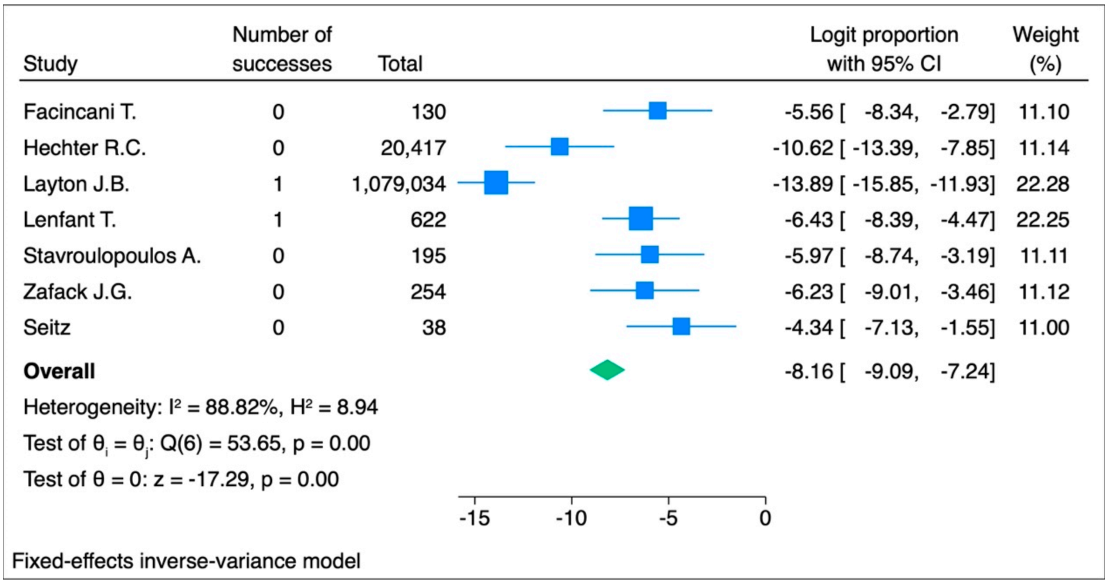

Supplement: Supplementary file 1 [file vaccines-13-00037-s001.zip › vaccines-3390244-supplementary.pdf]
